# Mitochondrial morphology is associated with respiratory chain uncoupling in autism spectrum disorder

**DOI:** 10.1038/s41398-021-01647-6

**Published:** 2021-10-13

**Authors:** Richard E. Frye, Loïc Lionnard, Indrapal Singh, Mohammad A. Karim, Hanane Chajra, Mathilde Frechet, Karima Kissa, Victor Racine, Amrit Ammanamanchi, Patrick John McCarty, Leanna Delhey, Marie Tippett, Shannon Rose, Abdel Aouacheria

**Affiliations:** 1 Phoenix Children’s Hospital, Phoenix, AZ USA; 2 University of Arizona College of Medicine – Phoenix, Phoenix, AZ USA; 3 Institut des Sciences de l’Evolution de Montpellier, UMR 5554 CNRS, UM, IRD, EPHE, Université de Montpellier, Place Eugène Bataillon, 34095 Montpellier cedex 05, France; 4 Clariant Active ingredients, 195 Route d’Espagne, 31036 Toulouse Cedex 1, France; 5 LPHI, CNRS, INSERM, Emergence of Haematopoietic Stem Cells and Cancer, Univ Montpellier, Montpellier, France; 6 QuantaCell SAS, 2 allée du Doyen Georges Brus, 33600 Pessac, France; 7 Arkansas Children’s Research Institute, Little Rock, AR USA

**Keywords:** Physiology, Molecular neuroscience

## Abstract

Autism spectrum disorder (ASD) is a neurodevelopmental disorder that is associated with unique changes in mitochondrial metabolism, including elevated respiration rates and morphological alterations. We examined electron transport chain (ETC) complex activity in fibroblasts derived from 18 children with ASD as well as mitochondrial morphology measurements in fibroblasts derived from the ASD participants and four typically developing controls. In ASD participants, symptoms severity was measured by the Social Responsiveness Scale and Aberrant Behavior Checklist. Mixed-model regression demonstrated that alterations in mitochondrial morphology were associated with both ETC Complex I+III and IV activity as well as the difference between ETC Complex I+III and IV activity. The subgroup of ASD participants with relative elevation in Complex IV activity demonstrated more typical mitochondrial morphology and milder ASD related symptoms. This study is limited by sample size given the invasive nature of obtaining fibroblasts from children. Furthermore, since mitochondrial function is heterogenous across tissues, the result may be specific to fibroblast respiration. Previous studies have separately described elevated ETC Complex IV activity and changes in mitochondrial morphology in cells derived from children with ASD but this is the first study to link these two findings in mitochondrial metabolism. The association between a difference in ETC complex I+III and IV activity and normal morphology suggests that mitochondrial in individuals with ASD may require ETC uncoupling to function optimally. Further studies should assess the molecular mechanisms behind these unique metabolic changes.

**Trial registration:** Protocols used in this study were registered in clinicaltrials.gov as NCT02000284 and NCT02003170.

## Introduction

Autism spectrum disorder (ASD) is a neurodevelopmental disorder that is characterized by deficits in social-communication along with restrictive and repetitive behaviors and interests [1]. Although diagnosing ASD can be complicated [2], the diagnosis is currently defined by a specific pattern of behavior which is outlined in the Diagnostic Statistical Manual of Mental Disorders Version 5 [1]. The Centers for Disease Control and Prevention funded Autism and Developmental Disabilities Monitoring Network currently estimates that ASD affects 1 in 54 children in the United States [3]. Despite decades of research, the etiology of ASD remains uncertain in most cases. Even though ASD is highly heritable, inherited genetic defects are rare [4] and most identified genetic mutations are de novo [5, 6]. For example, unbiased empirical clinical genetic evaluations show a low yield (~16%) even when using both chromosomal microarray and whole exome sequencing [7] and most of the time (69%) siblings with ASD have different de novo mutation [8].

Environmental exposures [9], especially during the prenatal period [10], may have a major role in modulating the risk of developing ASD. Indeed, it is now believed that ASD may arise from an interaction between genetic predisposition and environmental exposures [11, 12] including the availability of essential nutrients such as folate during the prenatal and preconception period [13]. For example, polymorphisms associated with one-carbon metabolism found in either the offspring [14] or mother [15] and autoantibodies that block folate transport into the brain and across the placenta [16] are associated with ASD, demonstrating that underlying weaknesses in the folate pathway might interact with environmental (folate intake) factors to increase the risk of ASD [13]. Likewise, multiple prenatal nutritional and toxicant exposures may disrupt physiology during prenatal development [10], particularly mitochondrial homeostasis [17]. Indeed, recent studies have linked long-term changes in mitochondrial function in children with ASD to prenatal environmental exposures [10, 17], including air pollution [18] and essential metals including Zn, Cu, and Mn [19, 20].

Best known for their role in adenosine triphosphate (ATP) production, mitochondria are also integrally involved in a variety of important cellular functions such as calcium buffering, redox regulation, apoptosis and inflammation [21–23]. Interestingly, studies have demonstrated that 30–50% of individuals with ASD demonstrate biomarkers of abnormal mitochondrial function [24, 25], while 80% of children with ASD may manifest abnormal electron transport chain (ETC) activity in immune cells [26, 27]. Compelling for ASD, many toxicants and nutrients associated with ASD risk can influence mitochondrial respiration [17].

Abnormalities in mitochondrial function have been linked to symptomatology in children with ASD. For example, a diagnosis of mitochondrial disease in children with ASD has been linked to fatigability, gastrointestinal disorders and unusual types of neurodevelopmental regression (NDR) including multiple regressions or regression later than commonly associated with ASD [28], seizures/epilepsy and motor delay [24] as well as other clinical abnormalities commonly associated with mitochondrial diseases such as developmental delays, ataxia, muscle weakness, peripheral neuropathy, endocrinology abnormalities, and failure to thrive [29].

One of the most compelling links between mitochondrial dysfunction and ASD symptomology is the subset of children with NDR. Indeed, the majority of children with ASD who are diagnosed with classic mitochondrial disease have a history of NDR, usually associated with a fever or inflammatory trigger [30]. Recently, NDR was also associated with elevated mitochondrial respiration in peripheral blood mononuclear cells (PBMCs) in children with ASD [31] and this mitochondrial abnormality has been linked to prenatal environmental influences [18, 19].

Unlike individuals diagnosed with classic mitochondrial disease where ETC activity is significantly depressed, some individuals with ASD have elevated ETC activity. Elevation in ETC Complex IV activity was first reported in ASD through muscle biopsy [32] and later confirmed in other tissues, including fibroblasts [33], buccal epithelium [34], brain [35] and lymphoblastoid cell lines (LCLs) [36]. Since ETC Complex IV is the ETC enzyme responsible for oxygen consumption, elevations in ETC Complex IV activity results in enhanced oxygen consumption also known as increased mitochondrial respiration. Parallel to elevations in ETC Complex IV activity, an LCL model of ASD shows elevation in mitochondrial respiration, about 200% of control LCLs, in a subset of ASD individuals [34, 37–43].

Most ETC complexes (I, III, IV) transport protons across the inner mitochondrial membrane in order to create a proton gradient which drives ETC Complex V, also referred to as ATP synthase, to make ATP. The ETC is the major source and target of reactive oxygen species (ROS) such as oxygen radicals. Oxidative stress occurs when ROS become elevated to the point where they can interfere with ETC function. In order to reduce ROS, the mitochondrion leaks protons back across the inner mitochondrial membrane, essentially decreasing the proton gradient and reducing mitochondrial efficiency. This proton leak uncouples the ETC complexes such that the activity across the ETC complexes is unequal. Interesting, the LCLs from individuals with ASD with elevated respiratory rates are associated with greater proton leak, uncoupling of the respiratory chain and superoxide elevation in the mitochondrial compartment [39, 43]. Consistent with this is a relative disassociation between ETC Complex I and IV activity in buccal epithelium from individuals with ASD, suggesting an uncoupling of the respiratory chain [44–46].

Preliminary studies have investigated the molecular regulation and consequences of elevated mitochondrial respiration in individuals with ASD. One study found that the LCLs with elevated mitochondrial respiration do not upregulate genes involved in mitochondrial dynamics and repair (SIRT1, SIRT3, PINK1, MFN2, DRP1, PGC1α, HIF1α) as compared to LCLs from children with ASD which have mitochondrial respiration similar to control LCLs [41]. Dysregulation of genes important for mitochondrial dynamics and repair is consistent with a recent study using nanometer-scale transmission electron microscopy which demonstrated morphological changes in individual mitochondria in fibroblasts from children with ASD and elevated mitochondrial respiration [47].

The discovery that genes involved in mitochondrial dynamics may be involved in variations in mitochondrial respiration in ASD is compelling. Mitochondria constantly undergo cycles of fission and fusion, dynamic processes which maintain mitochondrial health by eliminating dysfunctional mitochondria and repairing damaged mitochondria. Failure of these processes is associated with clinical disease and aging [48, 49]. Mitochondria demonstrate considerable variability in shape and size with morphology varying from long tubules and small spheres. Commonly thought of as an isolated static organelle, mitochondria are now known to form networks that may optimize their function and change subcellular positioning by moving along cytoskeletal tracks to reach sites of high-energy need [50]. Thus, understanding variation in mitochondrial morphology and their link to mitochondrial respiration can provide insight into the significance of mitochondrial function and dysfunction in ASD.

Only nanometer-scale electron microscopy has been used to examine mitochondrial morphology in ASD [47]. Imaging on a nanometer scale cannot quantitatively assess mitochondrial morphology in large numbers of mitochondria or understand mitochondrial positioning and branching in the entire, live cell. Indeed, laser confocal fluorescence microscopy techniques which image on a micrometer scale can measure mitochondrial morphometric characteristics of both individual mitochondria and networks of mitochondria as well as provide information regarding the cellular position of mitochondria [51]. Thus, this study aims to make the connection between quantitative measurements of morphological changes in the mitochondrial reticulum at the whole-cell level with specific changes in mitochondrial respiration in relation to ASD.

## Material and methods

Protocols used in this study were registered in clinicaltrials.gov as NCT02000284 and NCT02003170 and approved by the Institutional Review Board at the University of Arkansas for Medical Sciences (Little Rock, AR).

### Participants

Parents of participants provided written informed consent. All participants were recruited from the Arkansas Children’s Hospital Autism Multispecialty clinic directed by Dr Richard E Frye (first author).

The ASD diagnosis was documented by at least one of the following criteria: (i) a gold-standard diagnostic instrument such as the Autism Diagnostic Observation Schedule and/or Autism Diagnostic Interview-Revised (ADI-R); (ii) the state of Arkansas diagnostic standard, defined as agreement of a physician, psychologist and speech therapist who specializes in ASD; and/or (iii) Diagnostic Statistical Manual of Mental Disorders diagnosis by a physician along with standardized validated questionnaires including the Social Responsiveness Scale (SRS) [52, 53], the Social Communication Questionnaire [54–56] and the Autism Symptoms Questionnaire [57], all of which have excellent correspondence to gold-standard instruments, along with diagnosis confirmation by the Principal Investigator (first author). In our recent clinical trial [58], we found that methods (ii) and (iii) are consistent with the ADI-R diagnostic criteria for ASD.

In 17 of the 18 ASD participants, ASD symptoms were characterized using standardized instruments, specifically the caretaker completed Aberrant Behavior Checklist (ABC) and SRS [44, 58]. The ABC measures disruptive behaviors commonly associated with ASD and has convergent and divergent validity [59–61]. The SRS measures social skill deficits across five domains and corresponds well with gold-standard instruments [52, 53].

In general, fibroblast samples were obtained for clinical use and then transferred to the research laboratory. For individuals who underwent sedated procedures, most commonly muscle biopsy, the samples were obtained under sedation by the surgeon. For individuals that did not undergo other procedures, Dr Richard E Frye personally obtained the sample by punch biopsy with local anesthesia. Overall, all available fibroblast samples that were available at the time of analysis were included in this study. Demographics are given in Table 1. Average (SD) age was 7 years 0 months (3 years 7 months), with 72% Male, 78% White and 22% Asian, all without Hispanic ethnicity.

**Table 1:** Fibroblast Cell Lines Examined.

Cell line	Age	Gender	Race	Number of experiments	Number of cells	Number of microscope fields
*Autism spectrum disorder*						
AMC007	5yo	M	White	3	511	44
AMC027	5yo	F	White	3	328	43
AMC109	11yo	F	White	3	400	45
AMC147	6yo	M	White	3	280	45
AMC163	16yo	M	White	3	422	45
AMC234	8yo	F	White	3	370	45
AMC293	7yo	M	Asian	3	384	45
AMC315	6yo	M	White	3	327	45
AMC317	3yo	F	White	3	307	45
AMC410	4yo	M	Asian	3	579	45
AMC439	7yo	M	White	3	630	45
AMC440	3yo	M	White	3	315	45
AMC447	13yo	M	White	3	456	45
AMC507	7yo	M	Asian	3	1043	45
AMC525	5yo	F	White	3	712	45
AMC553	11yo	M	White	3	372	45
AMC565	4yo	M	Asian	3	955	45
AMC582	5yo	M	White	3	445	45
Avg (SD)	7y 0m (3y 7m)	72% Male	78% White	3 (0)	490 (226)	45 (0.5)
*Controls*						
GM01651	13yo	F	White	3	579	45
GM01864	11yo	M	White	3	202	45
GM02036	11yo	F	White	3	243	45
GM08398	8yo	M	White	3	666	50
Avg (SD)	10y 9m (2y 1m)	50% Male	100% White	3 (0)	423 (234)	46 (2.5)

Four control fibroblasts from children of slightly older age [Average (SD) age 10 years 9 months (2 years 1 month)], slightly less males [50% Male] and 100% White race without Hispanic ethnicity who did not manifest any known medical disease or genetic abnormalities were obtained from Coriell Institute for Medical Research (Camden, NJ). Although there are fewer control fibroblasts than ASD fibroblasts, there are several justifications for this. First, control fibroblasts from appropriate age (children) are limited in their availability due to ethical reasons since they require a skin biopsy which is a painful procedure. Second, the variability in mitochondrial function is much greater in individuals with ASD, so a greater number of samples are needed to adequately obtain a representative sample in those with ASD.

### Fibroblast processing and culture

Excised skin was placed immediately into 15 mL conical tube containing DMEM (1X) plus Glutamax with 15% fetal bovine serum, 4.5 g/L d-Glucose, 110 mg/ml Sodium Pyruvate and 1% penicillin-streptomycin. Samples were then transferred under sterile hood into separate 15 mL conical tubes containing 2 mL digestion media with DMEM, 20% FBS, 0.25% collagenase type I (Worthington-biochem.com; CLS-1; 235U/mg; lot 49E11273), 0.05% DNAse (Sigma Cat# DN25-100MG), and 1% penicillin-streptomycin and placed in upright position in an incubator at 37 °C overnight. The next day the conical tube was vortexed for 20 s to disrupt the skin, separate the epidermis and disintegrate the dermis. The outside of the 15 mL conical tube was sterilized and 3.0 mL of fibroblast culture media (DMEM + glucose, sodium pyruvate, and L-glutamine, 15% FBS, and 1% penicillin-streptomycin) was added. This mixture was pipetted up and down gently to mix thoroughly and then the contents were plated in a T25 tissue culture flask. The T25 culture flask was incubated and fibroblasts were split using 0.25% trypsin/EDTA when confluent.

### Mitochondrial respiratory measurements

Mitochondrial enzyme activity, including ETC and citrate synthase activities, was measured in fibroblasts derived from the ASD participants by one of the few standard clinical laboratories, Baylor Medical Genetics Laboratory (Houston, TX). Normative values (mean, standard deviation) from this laboratory are widely used and widely accepted to define normal and abnormal enzyme activity and used to diagnosis mitochondrial disease by physicians in the United States [62]. Such normative values have been used in previous studies of ETC activity in ASD [33, 63]. Briefly, the assays typically use spectrophotometry to quantify the resulting reaction when substrates are provided for a specific enzyme complex. The portion of ETC Complex I that is sensitive to rotenone (a complex I inhibitor) is also quantified. ETC Complex III activity is measured in combination with ETC Complex I and II for several reasons. First, the substrate for ETC Complex III is Ubiquinol, which is that product of ETC Complex I and II which reduces Ubiquinone to Ubiquinol. Thus, an effective manner for providing substrate to ETC Complex III is through testing along with ETC Complex I or II. Second, testing the combination of ETC Complex II and III together provides an index of CoQ10 deficiency. Third, ETC Complex I and III are part of an ETC super complex which functions most efficiently when the enzymes are functioning together. Examining their activity in combination can provide an index of the function of the super complex. Lastly, ETC Complex V is unstable in isolation, so it is less routinely measured and was not measured in this study.

For the current analysis, ETC activity was normalized into standard deviation (SD) units using these laboratory normative values. For each ETC complex, mean, SD and skewness of the ETC activity were calculated for the fibroblasts gathered from ASD participants. Skewness above or below 1 was considered significant. The number of cases outside the normative range was calculated as the number of cases above or below the 1.96 SD cutoff (i.e., below 2.5% and above 97.5% of the normal distribution). The significance of the number of cases outside the normal range was calculated using a binomial distribution with *p* = 0.975. α cutoff for significance was set to *p* < =0.001 given that 14 statistical comparisons were conducted (Bonferroni correction 0.05/14 = 0.004).

### Measurements of mitochondrial morphology

Measurement of mitochondrial morphology was conducted at the Institut des Sciences de l’Evolution de Montpellier (Montpellier, France) on both ASD and control fibroblasts. The laboratory was blind to the diagnosis of any specific sample. Fibroblasts were stained with MitoTracker Red FM, CellMask Green Plasma Membrane Stain (Thermofisher Scientific) and Hoechst 33342 (Merck) to concomitantly visualize mitochondria, cell membranes and nuclei. This procedure was adapted from our previously published protocol [64]. Following staining, cells were incubated in fresh, pre-warmed phenol-free medium at 37 °C and imaged live within an hour. Live-cell imaging was carried out under a Spinning Disc Nikon TI Andor CSU-W1 confocal microscope with objective set to ×60. Experiments were carried out in triplicate. A total 10,527 cells were examined over 992 microscope fields (Table 1).

**Fig. 1:**
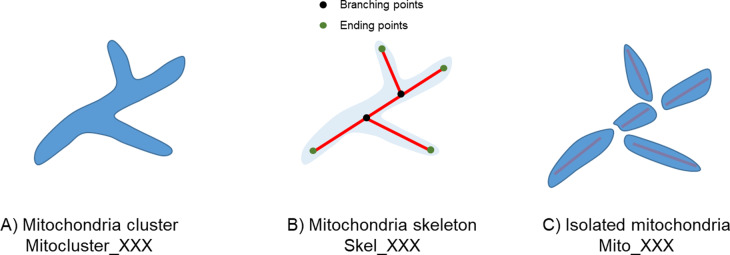
Process for obtaining mitochondrial morphology parameters. MITOTOUCH^®^ proprietary software identifies mitochondrial clusters, skeletonizes the clusters and divides them into individual mitochondria to derive both cluster and isolated mitochondrial metrics at the single cell level.

Images were processed using the MITOTOUCH^®^ proprietary software which will be described in detail elsewhere [65, 66]. Briefly, this computational image analysis program extracts a total of 31 features associated with geometrical (e.g., size, shape, connectivity) and non-geometrical cues (including texture and intensity) that provide a mathematical characterization of mitochondrial morphology and network organization in relation to cellular parameters (such as the shortest distance of mitochondria to the nucleus and to the cell membrane proximal region) (Table 2). As depicted in Fig. 1A, mitochondria clusters are identified and skeletonized. Branching points (black dots) and ending points (green dots) are identified from the skeleton (Fig. 1B). Branching points are used to divide clusters into mitochondrial fragments (Fig. 1C). Metrics are then derived from the cluster and mitochondrial fragments. Metrics represent cellular morphology, mitochondrial cluster morphology, mitochondrial skeleton morphology and mitochondrial cellular location together. Texture features that are usually not easy to capture through naked eye observation were also measured. For example, three measures of the fractal dimension capture the complexity of the mitochondrial structure at different resolutions. Likewise, the Euler number, also known as the Euler-Poincaré characteristic, measures curvature. The mean mitochondrial cluster count provides a relative index of the number of mitochondria per cell.

**Table 2:** Parameters for quantitative mitochondrial morphology measurements.

Feature	Definition
*Cellular parameters*	
Area	Total number of pixels occupied by the cell
Mean Intensity	Cellular mean intensity on cell channel
Max Intensity	Cellular max intensity on cell channel
Perimeter	Perimeter length in pixels
Compaction	Minor Axis Diameter/Major axis diameter on cell ellipse
Roundness	sqrt(4 × Area/pi)/ (Perimeter/4 × pi)
*Mitochondrial cluster parameters*	
Fractal8	Fractal dimension for a 8 × 8 pixels square
Fractal32	Fractal dimension for a 32 × 32 pixels square
Fractal64	Fractal dimension for a 64 × 64 pixels square
Count	Number of mitochondria cluster
Area	Mean of mitochondria cluster area
Elongation	Major Axis/Minor Axis Diameter on mitochondria cluster ellipse
Compaction	Minor Axis/Major Axis Diameter on mitochondria cluster ellipse
Roundness	Mean of sqrt(4 × Area/pi)/ (Perimeter/4 × π)
Euler number	Mean of Euler Number of each mitochondrial cluster
Mito Mean Intensity	Mean intensity of mitochondrial cluster
Mito Max Intensity	Mean of Max intensity of mitochondrial cluster
Perimeter	Mean perimeter of mitochondrial cluster
Solidity	Perimeter of the convex hull/Perimeter of the object
*Mitochondrial skeletonization parameters*	
Width	Mitochondria width compared to skeleton
Length	Total skeleton length
Branch Points	Number of skeleton branching points
Ending Points	Number of skeleton ending points
Branch Points Ratio	Branching points/(Branching points + ending points)
*Isolated mitochondria*	
Compaction	Minor Axis/Major Axis Diameter of isolated mitochondria
Elongation	Major Axis/Minor Axis Diameter of isolated mitochondria
Roundness	Sqrt(4 * Area/pi)/ (Perimeter/4 * pi) of isolated mitochondria
Length	Mitochondrial length
*Mitochondrial location*	
Distance To Membrane	Distance between mitochondria and cell membrane
Distance To Nuclei	Distance between mitochondria and nuclear envelope
Distance Ratio	DistToNuclei/(DistToCellMembrane + DistToNuclei)

### Statistical analysis

Statistical analysis was performed using PAWS Statistics 18 (SPSS Inc, Quarry Bay, HK). Graphs were produced using Excel version 16.0 (Microsoft Corp, Redmond, WA). A linear mixed-model was used to account for within-subject variation from repeated mitochondrial measurements on the same individual (replicates). Data were normally distributed.

Linear mixed-models expands the general linear model to allow the analysis of data with correlated and nonconstant variability. In other words, the mixed linear model not only models the means of the data but the variances and covariances as well. This is important for data in which the variance may be different across participants. The mixed-model does not require the variability to be the same across participants and allows this variability to be modeled as well as tested. This is important in measures of mitochondrial morphology where variation in morphology may differ across participants given the large number of mitochondria in the cell and the fact that mitochondria exhibit heteroplasmy—the notion that within the same cell, mitochondria can have genetic and functional variation. A detailed explanation of the mixed model is provided below.

The general mixed model is in matrix form

$${{{\mathrm{y}}}} = X\beta + {{{\mathrm{Z}}}}\gamma + \varepsilon$$

where *y* is the dependent variable, which in this case is the measure of mitochondrial morphology, *X* is the design matrix for the fixed effects, which in this case is the standardized ETC activity or group constants, β is a vector containing the parameters of the fixed effects, *Z* is the design matrix for the random effects, *γ* contains the parameters of the random effects and ε is the variance-covariance matrix of the model error. The key assumption of the mixed model is that both γ and ε have the expected value of zero (i.e., *E*(*γ*) = 0 and *E*(ε) = 0) and known covariance structure given by the matrixes Var (*γ*) and Var(ε). Random effects are modeled independently for each participant with a diagonal covariance structure assumed for the residuals. The linear mixed-model equation is solved by restricted maximum likelihood estimation with 100 maximum interaction, 5 maximum step-halvings, log-likelihood convergence of 0, parameter convergence of 0.000001, hessian convergence of 0 and singularity tolerance of 0.00000000001. In general, although not specifically reported for each model, the participant level random effects parameter of the y matrix was significant at the *p* <= 0.01 level as assessed by the Wald Z statistic.

For the regression examining relationship between mitochondrial morphological measurements and mitochondrial enzyme activity, the proportion of variance accounted for by the regression was determined by calculating the *R*^2^ using the equation below where n is the number of data points, y is the dependent variable and *ŷ* is the predicted value of the dependent variable. The *R*^2^ is represented as a percent and the *r* value is also calculated to provide an index of the effect size of the relationship. *r* values are a standardized measures of relationship strength with the following conventions: *r* of 0.1–0.3 is a small effect, *r* of 0.3–0.5 is a medium effect and *r* >= 0.50 is a large effect [67].

$$R^2 = 1 - \frac{{\mathop {\sum }\nolimits_{k = 1}^n \left( {y_i - \hat y_i} \right)^2}}{{\mathop {\sum }\nolimits_{k = 1}^n \left( {y_i - \bar y_i} \right)^2}}$$

To determine the relationship between mitochondrial morphological measurements and mitochondrial enzyme activity, ETC complex activities were considered independent variables. Only parameters with *p* < 0.001 were retained to account for multiple comparisons. Specifically, with 31 morphological measurements, Bonferroni correction suggests a *p* of 0.0016 (0.05/31). As described in the results, because of findings within the first analysis (Model 1), a transformed variable of ETC Complex IV minus ETC Complex I/III activity was created, and the models were rerun (Model 2).

Linear mixed-models were also used to examine the difference in mitochondrial morphology parameters between ASD vs Control groups. Since we found distinct variation in mitochondrial morphology depending on the relative elevation in ETC Complex IV activity as compared to ETC Complex I+III activity, we divided the ASD fibroblasts into two groups based on this difference in ETC complex activity to determine if the mitochondrial morphology of either group was similar to the mitochondrial morphology of the control fibroblasts. Comparison of the two ASD subgroups and controls were conducted for each morphology parameter. For analyses that demonstrated an overall significant group effect, the two ASD subgroups were compared to the control using planned post-hoc contrasts. An overall significant difference with Bonferroni correction of *p* = 0.0008 (0.05/(31*2)) was used. For two-tailed t-test with α = 0.0008, given the large number of observations, the difference between groups achieves a power of at least 95% down to a small effect size of Cohen’s *d* = 0.1. To provide an index of the magnitude of the difference between the two ASD group, the effect size as represented by Cohen’s *d* was calculated and interpreted as follows: 0.2 small effect, 0.5 medium effect and 0.8 large effect.

To investigate the relationship between mitochondrial morphology and ASD symptoms, two mitochondrial morphology measurements which differentiated the two ASD subgroups with different mitochondrial function the most, as based on the effect size, were selected. These morphology measurements were correlated with subscales of the ABC and SRS, two widely used measurements of ASD symptoms. Further, to determine whether the two ASD subgroups demonstrated distinct behavior, ABC and SRS scales were entered into a Fisher Discriminant Analysis to assess if a linear discriminant function could separate the two ASD subgroups.

## Results

In this study, mitochondrial enzyme activities from ASD fibroblasts are first compared to laboratory normative values. Next, an analysis of the relationship between ASD fibroblasts mitochondrial morphological measurements and mitochondrial complex activity is presented. Fibroblasts from individuals with ASD are then divided into two groups based on their mitochondrial enzyme activity, and the mitochondrial morphological measurements between these two groups are compared to the morphological measurements from the control fibroblasts. Lastly, the relationship between ASD behavior and selected morphological measurements and the two ASD groups is investigated.

### ETC complex activity in ASD fibroblasts

**Fig. 2:**
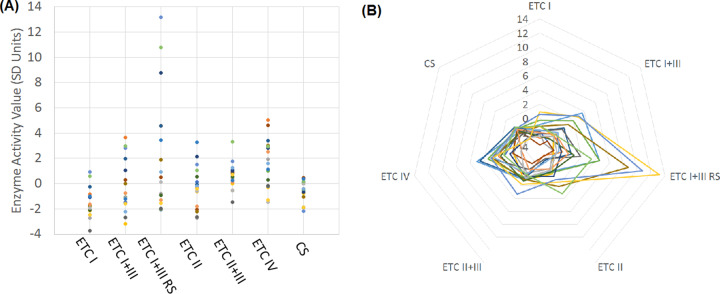
Electronic transport chain activity of autism spectrum disorder fibroblast cell lines. Standardized mitochondrial enzyme activity from 18 ASD fibroblasts represented in (A) linear graph and (B) radar plot. Green box represents normative range (±1.96 SD). ETC = Electron transport chain; CS = Citrate Synthase.

Table 3 outlines the laboratory normative mean and SD for fibroblast mitochondrial enzyme activities, along with the mean and SD for the fibroblasts obtained from the ASD participants. To examine the ASD participant values relative to the normative control values and to equalize the activity scales between the different enzymes, enzyme activity was standardized to the normative control values [i.e., Standardized Value = (ASD Value−Normative Mean)/Normative SD]. Figure 2 provides standardized enzyme activity values in graphical form. Table 4 outlines the standardized mitochondrial enzyme activity mean, SD and skewness.

**Table 3:** Electron transport chain complex activity in Fibroblasts for clinical normative reference range and for fibroblasts derived from children with autism spectrum disorder.

	Normative		ASD Participants	
Enzyme (nmoles/min/mg protein)	Mean	Standard deviation	Mean	Standard deviation
ETC Complex I	1026	196	730.74	224.36
ETC Complex I+III	190	25.0	180.71	51.90
ETC Complex I+III RS	56.5	15.0	86.73	70.38
ETC Complex II	6.98	0.98	6.33	2.25
ETC Complex II+III	4.00	0.90	4.32	1.41
ETC Complex IV	17.1	4.10	23.91	7.86
Citrate Synthase	67.8	14.1	61.47	11.58

**Table 4:** Electron transport chain complex activity (normalized) in fibroblasts derived from children with autism spectrum disorder.

Enzyme	Mean	Standard deviation	Skewness	Below normal	Above normal
ETC Complex I	−1.51	1.45	0.45	33% (6/18)^****^	0% (0/18)
ETC Complex I+III	−0.37	2.08	0.62	22% (4/18)^**^	17% (3/18)
ETC Complex I+III RS	***2.02***	4.69	***1.18***	11% (2/18)	33% (6/18)^****^
ETC Complex II	−0.29	1.68	0.34	22% (4/17)^**^	11% (2/17)
ETC Complex II+III	0.63	1.05	0.49	0% (0/17)	6% (1/17)
ETC Complex IV	1.66	1.92	0.00	0% (0/17)	44% (8/18)^*****^
Citrate Synthase	−0.44	0.82	−0.90	6% (1/17)	0% (0/17)

Values in bold, underline and italic are outside the normal range. Since these are normalized values, they are in standard deviation units.

**p < =0.001; ***p < =0.0001; ****p < =0.00001; *****p < =0.000001.

ETC Complex I demonstrated a low mean with a relatively normal SD and skewness suggesting that the distribution was depressed across the group of ASD individuals, consistent with Fig. 2A which demonstrates a downward shift in the distribution. ETC Complex I+III demonstrated a relatively normal mean and skewness with an increased SD, suggesting increased variability, consistent with Fig. 2A. ETC Complex I+III RS demonstrated an increased SD with positive skewness indicating unique cases with elevated activity, producing a long distribution tail. In other words, the ASD participants as a group did not have an overall average increase in ETC Complex I+III RS activity but rather there were a few unique individuals, perhaps a subgroup, that represented elevated activity in this mitochondrial enzyme, consistent with Fig. 2A, B. Complex II demonstrated a relatively normal mean, SD and skewness. Complex IV demonstrated an elevated mean with a relatively normal SD and skewness suggesting that the distribution was increased across the group of ASD individuals equally, consistent with Fig. 2A.

Table 4 outlines the number of cases outside the normative range as well as the significance of this number as calculated using a Binomial distribution. Three ETC Complexes had more cases below the normal range than would be expected by chance. ETC Complex I demonstrated below normal activity in 33% of the ASD participants while ETC Complex I+III and II demonstrated below normal activity in 22% of the ASD participants. Two ETC Complex demonstrated more cases above the normal range than would be expected by chance. ETC Complex I+III RS demonstrated above normal activity in 33% of the ASD participants while ETC Complex IV demonstrated above normal activity in 44% of the ASD participants.

### Relationship between mitochondrial morphology and ETC complex activity

A mixed-model regression was used to determine the relationship between mitochondrial enzyme activity and mitochondrial morphology. Parameters for enzymes found to be non-significant (cutoff *p* < 0.001) were eliminated from the model. Two models were analyzed. Model 1 included all mitochondrial enzyme variables. Model 2 added the transformed variable of Complex IV activity minus Complex I+III activity because of findings from the first model. F statistics for each significant mitochondrial enzyme activity parameter is provided in Supplementary Table 1 while parameter values are provided in Table 5.

**Table 5:** Coefficients for two mixed-models [Mean (Standard Error)] examining the association between electron transport chain (ETC) complex activity and morphology in fibroblasts derived from children with autism spectrum disorder. R^2^ and r values for model 2 are also depicted to provide a representation of the effect size of the regression.

	Model 1		Model 2	
			Complex IV minus I/III	
Morphological measurement	Complex I+III	Complex IV	Parameter value	*R*^2^/r
**Cellular parameters**				
***Area***	−58,300 (5628)	60,553 (5080)	59,357 (1987)	33.7%/0.58
***Max Intensity***	−490.2 (61.0)	439.1 (54.7)	462 (18.2)	31.2%/0.56
***Perimeter***	−951.5 (88.2)	966.8 (79.6)	957 (31.2)	27.8%/0.53
Compaction	0.064 (0.006)	−0.059 (0.006)	−0.0611 (0.003)	12.4%/0.35
Roundness	0.023 (0.002)	−0.021 (0.002)	−0.022 (0.001)	20.4%/0.45
**Mitochondrial cluster parameters**				
***Fractal 8***	−0.106 (0.013)	0.094 (0.012)	0.099 (0.005)	29.6%/0.54
***Fractal 32***	−0.218 (0.027)	0.197 (0.024)	0.206 (0.011)	32.0%/0.56
***Fractal 64***	−0.210 (0.090)	0193 (0.023)	0.200 (0.010)	34.8%/0.59
Count	−19.41 (2.43)	21.04 (2.24)	20.2 (1.2)	21.0%/0.46
***Area***	−6.50 (1.17)	5.42 (1.07)	5.91 (0.5)	44.9%/0.67
***Compaction***	0.012 (0.002)	−0.011 (0.001)	−0.012 (0.001)	26.5%/0.51
***Elongation***	−0.126 (0.014)	0.120 (0.013)	0.122 (0.008)	32.3%/0.57
***Roundness***	0.0027 (0.0004)	−0.0022 (0.0004)	−0.0024 (0.0002)	34.0%/0.58
***Mito Mean Intensity***	854 (29.8)		13.04 (3.84)	30.8%/0.56
***Mito Max Intensity***	−76.1 (11.1)		40.00 (5.62)	30.1%/0.55
***Perimeter***	−2.38 (0.47)	1.89 (0.43)	2.11 (0.20)	45.0%/0.67
***Solidity***	0.0034 (0.0010)		−0.019 (0.0005)	36.8%/0.61
**Mitochondrial skeletonization parameters**				
***Skeletal Width***	−0.047 (0.007)	0.048 (0.007)	0.048 (0.003)	45.8%/0.68
Skeletal Length	−478 (57)	484 (53)	−1083 (176)	18.2%/0.43
Branch Points	−24.0 (3.26)	25.0 (3.12)	24.3 (1.76)	18.7%/0.43
Ending Points	−61.76 (7.95)	66.08 (7.34)	63.6 (3.97)	20.0%/0.45
**Isolated mitochondria**				
Compaction	0.007 (0.002)	−0.006 (0.001)	−0.0067 (0.0009)	23.5%/0.48
***Elongation***	−0.181 (0.022)	0.170 (0.020)	0.174 (0.0105)	37.5%/0.61
Roundness	0.0055 (0.0009)	−0.0047 (0.0009)	−0.0050 (0.0005)	19.3%/0.44
***Length***	−0.414 (0.051)	0.382 (0.047)	0.395 (0.025)	37.1%/0.61
**Mitochondrial location**				
***Cell Membrane***	−6.11 (1.13)	6.44 (1.02)	6.26 (0.41)	51.1%/0.72
***Nucleus***	−17.17 (1.71)	17.48 (2.48)	19.5 (0.98)	30.2%/0.55

Model 1 examined individuals ETC complex activity while Model 2 examined a transformed variable which represents the difference between ETC complex I/III and IV. All coefficients were significant *p* < 0.0001. The morphological measurements with large effect sizes are bold, italic and underlined for emphasis.

*R*^2^ was rather consistent for morphological measures across both models (Supplementary Table 1) with the percent variance accounted for varying from 12.4% to 51.1% with an average across all models of 30.5%. Corresponding r values varied, ranging from 0.35 (medium effect) to 0.72 (large effect) with an average of 0.55 (large effect). R^2^ and r values for Model 2 are provided in Table 5. Morphological measurements with large effect sizes are bold, italic and underlined.

**Fig. 3:**
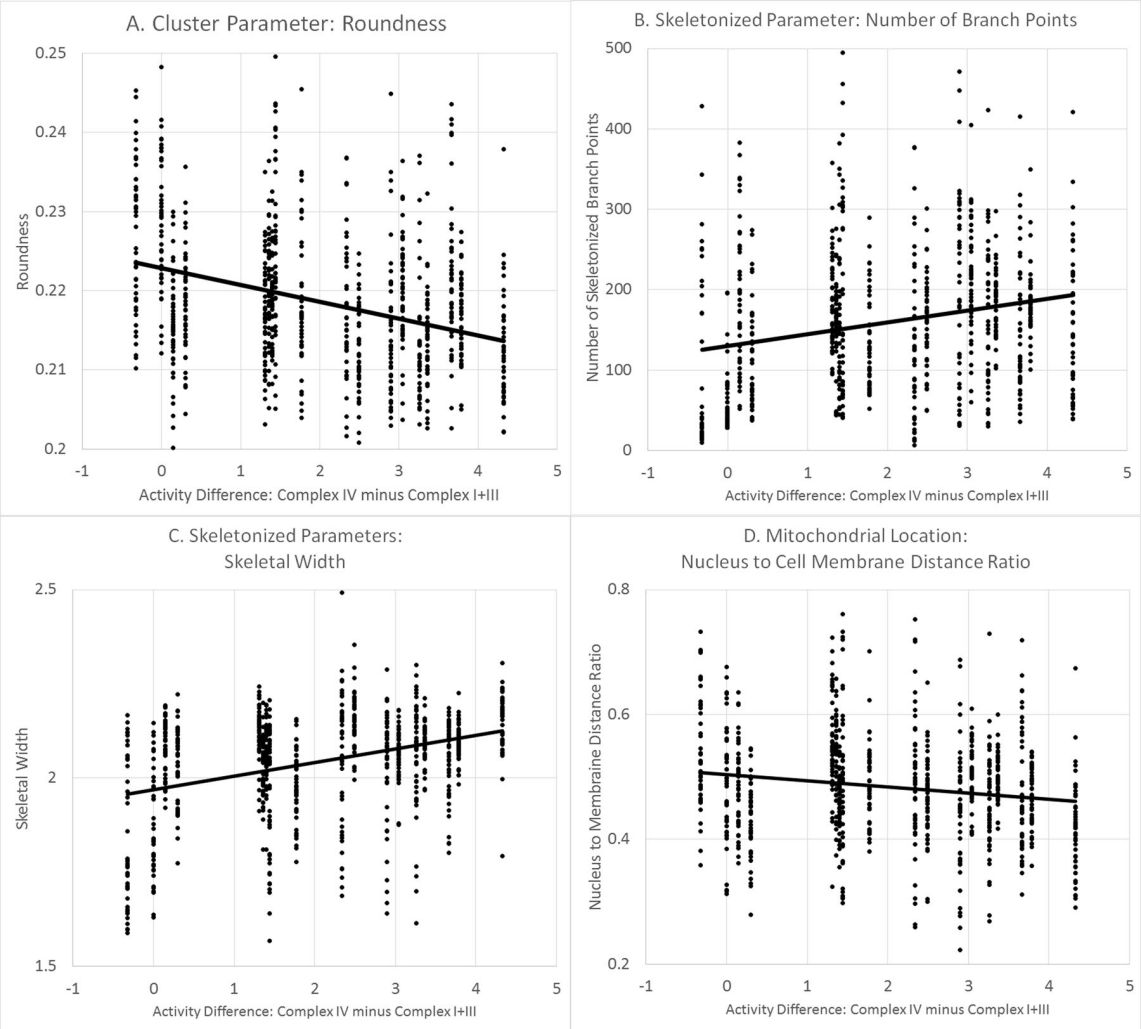
Relationship between mitochondrial respiration and morphology in autism spectrum disorder fibroblast cell line. Examples of the relationship between mitochondrial morphological parameters and mitochondrial ETC complex activity for fibroblasts derived from children with ASD. Individual morphological measurements from each cell examined are depicted.

For Model 1, the majority of the morphological measurements were found to be significantly associated with ETC Complex I+III and Complex IV activity with the relationships between morphology and mitochondrial enzyme activity opposite for these two ETC complexes. For example, higher ETC Complex I+III activity was associated with a smaller cellular area, maximum intensity and perimeter and a higher cellular mean intensity, compaction and roundness, whereas a higher ETC Complex IV activity was associated with a larger cellular area, maximum intensity and perimeter and a lower cellular mean intensity, compaction and roundness. Thus, the analyses using Model 1 suggested that, in general, activity of ETC Complex I+III and ETC Complex IV had an opposite association with cellular and mitochondrial morphology. Thus, a transformed variable which represented that activity in ETC Complex IV relative to ETC Complex I+III was created to determine if morphology was driven by the activity difference. Analysis using Model 2 demonstrated that the transformed variable was highly significant and accounted for the variance of ETC Complex IV and ETC Complex I+III activity parameters (See Table 5). Examples of the relationships between enzyme activity and mitochondrial morphology are shown in Fig. 3. Parameters from each parameter group with the largest effect sizes were chosen for display.

### Mitochondrial morphological measurements compared to controls

The above analysis suggests that the ASD fibroblasts have variation in cellular and mitochondrial morphology associated with ETC complex activity. To understand which of these variations was more like controls, ASD participants were split into two subgroups, a subgroup with relatively equal ETC Complex IV and I+III activities and a subgroup with higher activity in Complex IV relative to Complex I+III. An analysis of the subgroup values relative to controls was conducted (Table 6).

**Table 6:**
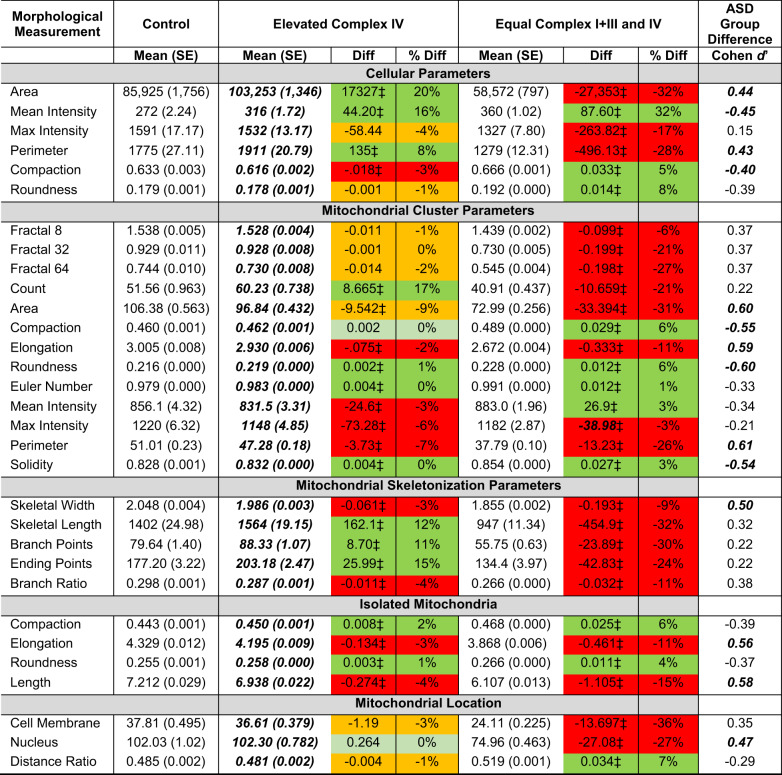
Means (SE = Standard Error) of morphological measurements for three groups, control fibroblasts, ASD fibroblasts with elevated Complex IV activity relative to Complex I+III and ASD fibroblasts with relatively equal Complex IV and I+III activities.

The mean and percent difference between the ASD subgroups and control group is provided. The subgroup mean that is closer to the control values has bold and italic values.

^‡^*p* < 0.0008 which is the threshold based on Bonferroni correction. The difference between the ASD groups is also represented by the Cohen *d*’ effect size with medium effect sizes in bold and italic. Color code: mean is statistically significantly higher than control (dark green); mean is higher than control but did not reach statistical significance (light green); mean is statistically significantly lower than control (red); mean is lower than control but did not reach statistical significance (orange).

As seen in Table 6, except for the Cluster parameter Maximum Intensity, the morphological measurements from the control fibroblasts were closer to fibroblasts with greater ETC Complex IV activity relative to Complex I+III activity as demonstrated by the bold and italic mean. In fact, the mean difference between the controls and the fibroblasts with greater ETC Complex IV activity relative to Complex I+III activity is 2 to 10 times smaller as compared to the mean difference between the controls and the fibroblasts with relatively equal ETC Complex IV and I+III activities. Furthermore, the difference between the controls and the fibroblasts with relatively equal ETC Complex IV and I+III activities was statistically significant for all the morphological measurements. In contrast, the differences in the morphological measurements between controls and the fibroblasts with greater ETC Complex IV activity relative to Complex I+III activity was not significant for Cellular Maximum Intensity and Cellular Roundness, Mitochondrial Cluster measurements Fractal 8, Fractal 32, Fractal 64, and Compaction and Mitochondrial Location parameters representing distance from Cell Membrane, Distance from Nucleus and Distance Ratio. Given the excellent power of the analysis, these non-significant mitochondrial morphology differences between the controls and the ASD subgroup with relatively high ETC Complex IV activity can be considered equivalent.

**Fig. 4:**
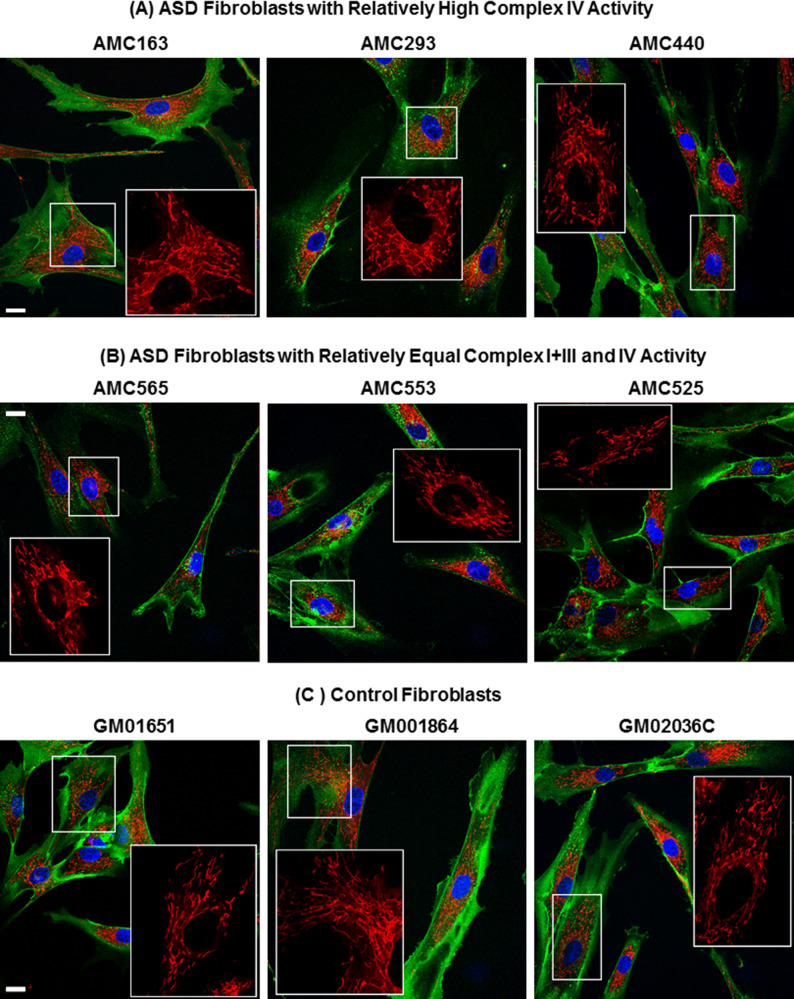
Images of mitochondrial morphology in fibroblast cell lines. Fibroblasts from patients with (**A**, **B**) autism spectrum disorder or from (**C**) control individuals. Mitochondria are highlighted in red, the cell membrane in green and nuclei in blue. **A** Top panels: fibroblasts from three ASD patients with relatively increased Electron Transport Chain Complex IV activity. **B** Middle Panels: fibroblasts from three individuals with autism spectrum disorder with roughly similar Electron Transport Chain Complex I+III RS and Complex IV activity. **C** Bottom Panels: fibroblasts from three control individuals. Scale bar: 15 µm. In each case, the inset is a ×2 magnification of a region-of-interest (indicated by a white box) showing the mitochondrial compartment. Consistent with the morphological measurements, mitochondria present in cells from ASD patients with relatively increased Electron Transport Chain Complex IV activity are found in larger cells and tend to be more abundant, more clustered and more branched. In contrast, mitochondria present in cells from ASD patients with relatively similar Electron Transport Chain Complex I+III RS and Complex IV activity tend to form more compact clusters of less complex geometry and appear to be more uniformly distributed throughout smaller cells.

Figure 4 depict images of mitochondria in fibroblasts with relatively higher ETC Complex IV activity (top panels), relatively similar ETC Complex I+III and IV activities (middle panels) and control fibroblasts (bottom panels), respectively. As can be seen, fibroblasts with relatively elevated ETC Complex IV activity share more resemblance to control fibroblasts in their mitochondrial (network) morphology than fibroblasts with relatively equal ETC Complex IV and I+III activities.

**Fig. 5:**
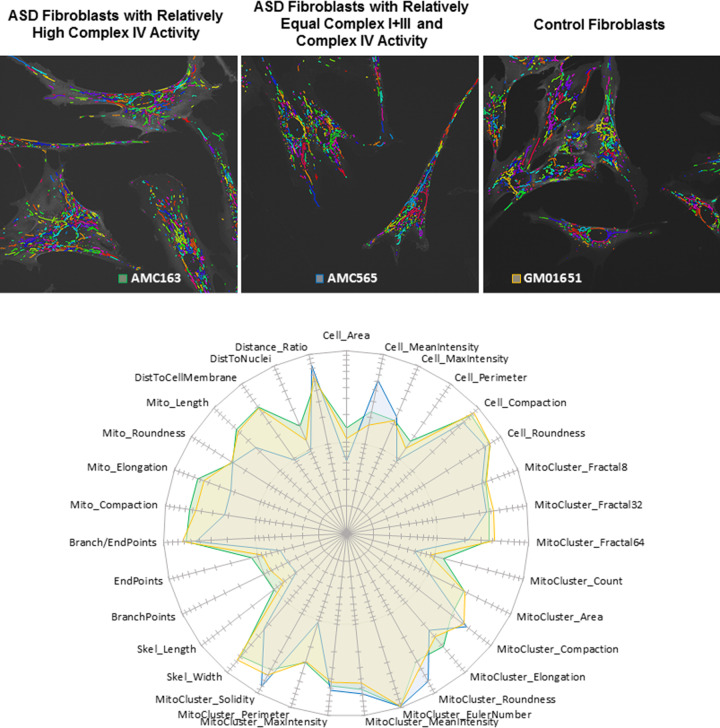
Radar-chart distribution of normalized morphological features obtained for representative fibroblasts from patients with autism spectrum disorder or from control individuals. Three representative microscopic fields are shown that correspond to control fibroblasts (GM01651), ASD fibroblasts with elevated Complex IV activity relative to Complex I+III (AMC163) and ASD fibroblasts with relatively equal Complex IV and I+III activities (AMC565). The segmented images (top panels) were randomly colorized for illustrative purposes. Parameter values *V*_p_ (expressed as arbitrary units) were obtained using the MITOTOUCH^®^ software. Minimum value (Vmin) and maximum value (Vmax) were derived for each parameter based on the entire data set. Values *V*_p_ were normalized (to give *V*_n_) by applying the following formula: *V*_n_ = (*V*_p_ – *V*_min_)/(*V*_max_ – *V*_min_) and *V*_n_ values were reported on the radar chart (bottom panel). Fibroblasts with relatively increased Complex IV activity (green line) have their morphological feature space more similar to that of control fibroblasts (orange) (for 29 parameters out of 31) whereas the morphological feature space of fibroblasts with relatively equal ETC Complex I+III and IV activities (blue) appear to be slightly distant.

Table 7 outlines the morphological parameters that have higher values in the mitochondria with relatively equal ETC Complex I+III and IV activities and those with relatively elevated ETC Complex IV activity. Fibroblasts with mitochondria exhibiting increased ETC Complex IV activity tend to be larger (having greater area and perimeter with a higher mean staining intensity) and display more clusters of reticulated mitochondria (with more branch points). In contrast, fibroblasts with mitochondria with similar ETC Complex I+III and IV activities have more compact and rounder cellular and mitochondrial morphology with mitochondrial clusters having less complex geometry and more uniform distribution within cells. Results from the morphological measurements were roughly consistent with the confocal microscopy data (Fig. 4), demonstrating the usefulness of objective quantitative evaluation of morphological parameters for image interpretation. Last, we used radar graphs (Fig. 5) to synthesize (in the form of ‘phenotypic signatures’) the multivariate data from three representative samples (bottom panel), paired with their respective segmented images (top panels). This visual display further underscores the fact that fibroblasts with relatively increased Complex IV activity have a mitochondrial morphology more similar to that of control fibroblasts whereas those with relatively equal ETC Complex I+III and IV activities tend to differ from controls in most of their morphological parameter values.

**Table 7:** Morphological parameters from Table 6 which have higher values organized by ETC complex IV activity.

Higher for mitochondria with elevated ETC complex IV activity		Higher for mitochondria with similar ETC complex IV and I+III activity	
Cellular	Skeletonization	Cellular	
***Area***	***Width*** and Length	Mean Intensity	
***Max Intensity***	Branch Points	***Compaction***	Isolated
***Perimeter***	Ending Points	Roundness	Compaction
Cluster	Isolated	Cluster	Roundness
Fractal 8,32,64	***Elongation***	***Compaction***	Location
Count, ***Area***	***Length***	***Roundness***	Distance Ratio
***Elongation***	Location	***Solidity***	
***Perimeter***	Cell Membrane	Mean & Max Intensity	
	***Nucleus***	Euler Number	

Parameters which demonstrate at least medium effect sizes (as calculated by Cohen ***d***’) are displayed in bold and italic.

### Association of mitochondrial morphological measurements with ASD symptoms

**Fig. 6:**
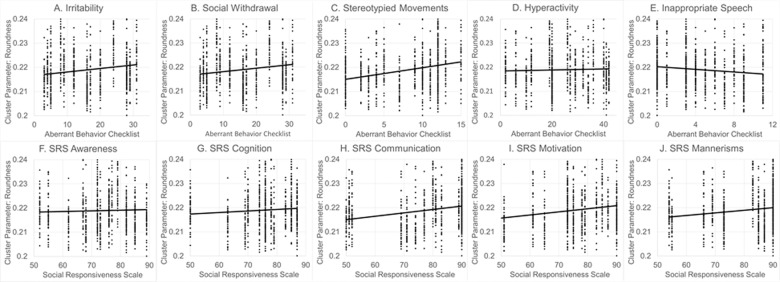
The relationship between ASD symptoms and behaviors for mitochondrial cluster roundness. Both (**B**) social withdrawal and (**C**) stereotyped movements on the aberrant behavior checklist (ABC) were significantly worse with increased mitochondrial cluster roundness.

**Fig. 7:**
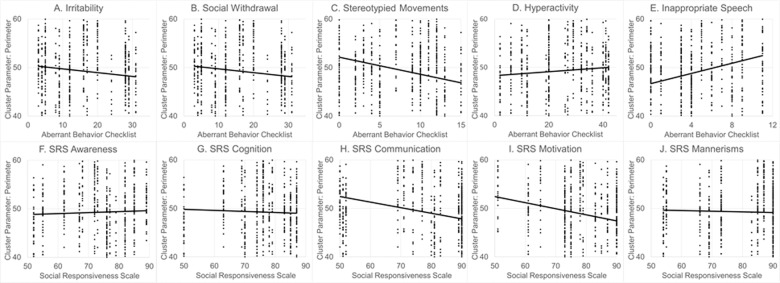
The relationship between ASD symptoms and behaviors for mitochondrial cluster perimeter. Both (**B**) Social Withdrawal and (**C**) Stereotyped Movements on the Aberrant Behavior Checklist (ABC) were significantly better with higher mitochondrial cluster perimeter.

To investigate the relationship between mitochondrial morphology and ASD symptoms, the mitochondrial cluster perimeter was selected as it had the largest effect size and was higher for the ASD subgroup with relatively greater Complex IV activity and mitochondrial cluster roundness was selected since it had the second highest effect size and was higher for the ASD subgroup with relative equal Complex I+III and Complex IV activity . As seen in Fig. 6, higher mitochondrial cluster roundness was significantly associated with more severe deficits in Social Withdrawal [*F*(1,15.3) = 8.50, *p* = 0.01; *R*^2^ 32%, *r* = 0.57; Fig. 6B] and Stereotyped Movements [*F*(1,15.1)=8.86, *R*^2^ 32%, *r* = 0.57; *p* < 0.01; Fig. 6C]. As seen in Fig. 7, higher mitochondrial cluster perimeter was significantly assocaited with better Social Withdrawal [*F*(1,15.0) = 6.33, *p* = 0.02; *R*^2^ 43%, *r* = 0.66; Fig. 7B] and Stereotyped Movement [*F*(1,15.0) = 4.33, *p* = 0.05; *R*^2^ 43%, *r* = 0.66; Fig. 7C].

**Fig. 8:**
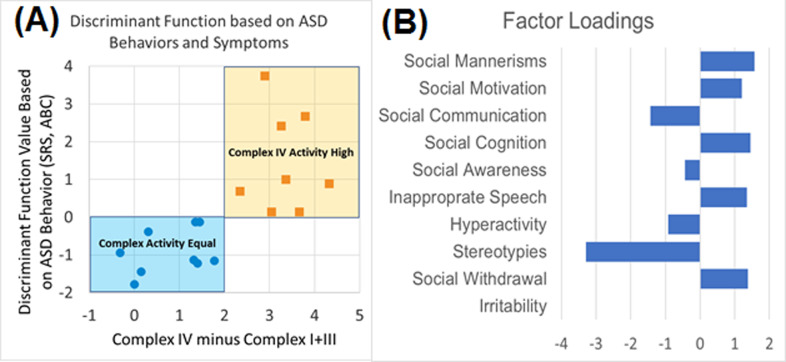
The Discriminant Function developed from scales of ASD symptoms and behavior. **A** Function separates ASD individuals into the two mitochondrial activity subgroups. **B** Loadings (influence) of individual ASD symptom and behavior scales.

Given the relationship between mitochondrial function and morphology and the relationship outlined above between morphology and behavior, it would be expected that differences in behavior would correspond to the ASD mitochondrial function subgroups. Hence, Fisher Discrimant analysis was used to determine if symptoms could classify cases into the two groups. Fisher Discriminant analysis created a linear function which classifed the cases with a 100% accuracy (Fig. 8A). Loadings indicated that more negative scores were driven by stereotypies and deficits in social communication while more positive scores were driven by impairments in social cognition, social motivation and social withdrawal.

## Discussion

This study examined the mitochondrial morphology of fibroblasts from individuals with ASD as well as mitochondrial enzyme activity, particularly activity of the ETC complexes. Mitochondrial features were examined using a computerized, automated method [65, 66] that objectively captured subtle differences in morphology and network connectivity from confocal fluorescence images. Both significant depression in ETC complex activity, particularly ETC Complex I activity, as well as elevation in ETC complex activity, particularly of Complex IV, was found; these changes are consistent with previous findings on mitochondrial ETC Complex activity in ASD, as discussed below. ETC Complex I+III and IV activities were both found to be significantly associated with variations in mitochondrial morphology albeit in opposite directions. Examining the difference between activities in these two ETC complexes revealed that this difference was distributed over a continuum from no difference to ETC Complex IV having a higher activity than ETC Complex I+III. This variation in activity was associated with mitochondrial morphology. When comparing the two groups of ASD fibroblasts, the fibroblasts with ETC Complex IV activity greater than ETC Complex I+III activity were larger and contained more clusters of mitochondria which, on average, were more branched while fibroblasts with relatively similar ETC Complex I+III and Complex IV activity tended to be smaller cells and contained less reticulated and less complex mitochondria that were also smaller in size and more evenly distributed throughout the cell. ASD fibroblasts with a relatively higher ETC Complex IV activity relative to ETC Complex I+III activity were most like the control fibroblasts in their mitochondrial morphology. Mitochondrial morphology was also found to be related to ASD symptoms and behavior.

Abnormal mitochondrial function is one of the most prevalent metabolic disorders associated with ASD, although the type of mitochondrial abnormality seems to vary considerably from study to study. A meta-analysis found that the prevalence of classic mitochondrial disease in ASD is approximately 5% [24]. Consistent with the findings of this study, the meta-analysis found that ETC Complex I deficiency was the most common respiratory deficiency in those with classically defined mitochondrial disease [24]. Interestingly, this meta-analysis also found that genetic defects could explain the mitochondrial abnormalities in only about 25% of the cases, suggesting that the great majority of cases were not caused by simple known genetic defects [24].

In contrast to a 5% prevalence of classic mitochondrial disease, studies that have measured ETC activity in individuals with ASD have reported much higher prevalence of abnormal mitochondrial enzyme activity. Measurements of ETC Complex I, IV and Citrate Synthase activity from buccal samples found activity outside of the normal range in 62–65% of the ASD participants in two studies [44, 45] while two other studies measuring ETC activity in lymphocytes and granulocytes reported that 80% of the ASD samples had enzyme activity outside of the normal range [26, 27]. Supportive of a high prevalence of abnormal mitochondrial enzyme activity is the fact that biomarkers of mitochondrial dysfunction are also reported to be abnormal in 8–47% of individuals with ASD [24, 68].

The discrepancy between the prevalence of classic mitochondrial disease and physiological measurement of mitochondrial dysfunction can be explained by the findings of atypical (i.e., different from classical mitochondrial disease) mitochondrial dysfunction in individuals with ASD. Several variations of unique abnormalities in mitochondrial dysfunction have been reported. Abnormalities in fatty-acid oxidation metabolism have been associated with ASD in many studies [69]. One study reported unique elevations in short and long chain acyl-carnitine in a subset of ASD patients in a large case series [25] with a follow-up study further estimating the prevalence of these abnormalities to be approximately 17% and further defining alterations in mitochondrial enzyme activity, most notably a depression in ETC Complex II activity in fibroblasts [33]. Thus, the depression in ETC Complex II activity in 22% of the patients as found in the current study is consistent with these previous observations.

Another type of mitochondrial dysfunction associated with ASD is manifested by increased activity of the mitochondrial respiratory chain, particularly an elevation in ETC Complex IV activity, also consistent with the findings of our study. The first report of significant elevation in ETC Complex IV activity was a case series of five ASD patients with muscle ETC Complex IV activity about 200% of normal [32]. Subsequently, associations of ASD with significantly high ETC Complex IV activity has been reported in buccal cells [44], LCLs [36] and fresh frozen post-mortem superior temporal gyrus [35]. Elevations in mitochondrial respiration, about 200% higher than control LCLs, has been shown to affect approximately 1/3 of ASD LCLs. These ASD LCLs can be divided into two subgroups depending on their mitochondrial respiratory rates: those with normal bioenergetics (AD-N) and those with atypical bioenergetics because of elevated respiratory rates (AD-A) [39]. This pattern of abnormal respiration in this subset of LCLs has been confirmed over eight studies [34, 37–43]. Interestingly, ASD LCLs with normal bioenergetics (i.e., AD-N LCLs) were found to upregulate genes associated with mitophagy including PINK1, MNF2, SIRT3, DNM1L, HIF1α and PGC1α. In contrast, ASD LCLs with elevated respiratory rates (i.e., AD-A LCLs) did not upregulate these genes involved in mitochondrial dynamics, repair and resistance to physiological stress [41]. As ETC Complex IV is the source of oxygen consumption in the ETC, the fact that mitochondrial morphology in the fibroblasts with relatively elevated Complex IV activity is similar to controls is consistent with this previous gene expression data demonstrating a more normal-like gene expression signature of the genes involved in mitochondrial morphology.

The finding that elevated ETC Complex IV activity relative to ETC Complex I+III rather than an absolute elevation in ETC Complex IV activity itself was related to maintaining normal mitochondrial morphology suggests that it is the uncoupling of the respiratory chain that is important to maintain normal mitochondrial morphology. This apparent uncoupling of the respiratory chain is consistent with other research on mitochondrial abnormalities associated with ASD. In fact, uncoupling of the respiratory chain by increasing proton leak through the inner membrane may be associated with the ASD phenotype [17]. An increase in proton leak respiration has been reported in ASD LCLs [68, 70]. An increase in Uncoupling Protein 2 gene expression [41, 43] and protein concentration [39] has been reported in ASD LCLs with elevated respiratory rates (i.e., AD-A LCLs). Adenine Nucleotide Translocator, which has a significant role in the regulation of inner mitochondrial membrane proton leak, shows increased expression when heteroplasmic levels of the mtDNA 3243A>G mutation is within the range associated with ASD [71] and a mutation in the ANT2 gene is associated with non-syndromic intellectual disability with ASD [72]. Lastly, the Fragile X syndrome mouse demonstrates proton leak through a dysfunctional ETC Complex IV and this increased leak has been shown to directly affect synaptic growth in this mouse model [73]. Thus, although proton leak could be a compensatory mechanism for controlling mitochondrial ROS, it may inhibit synaptic development.

The findings from this study may have significant treatment implications. One study on ASD LCLs with elevated respiratory rates (AD-A LCLs) demonstrated that activation of the S6K1 pathway may have been inhibiting the expression of genes involved in the regulation of mitochondrial morphology and response to cellular stress [41]. Since the S6K1 pathway is downstream from the mammalian target of rapamycin complex 1 (mTORC1), involvement of S6K1 was demonstrated by showing that low-dose rapamycin normalized the elevated respiratory rates in the AD-A LCLs [41]. Additional studies in MTORopathies demonstrate the importance of MTORC1 hyperactivity inhibiting mitochondrial dynamics in neurons [74]. Parallel to these cellular in vitro studies, several clinical trials have examined the effect of mTOR inhibitors on ASD symptoms. One case report [75] and series [76] suggested that ASD symptoms improved in 6 patients with ASD and tuberous sclerosis complex (TSC) with everolimus treatment. However, the two controlled clinical trials that have evaluated everolimus in children with tuberous sclerosis complex only found marginal positive effect in one trial [77] and no benefit in another [78].

PGC1α is not upregulated in LCLs from ASD individuals with elevated mitochondrial respiratory rates (AD-A) but is in LCLs from ASD with normal respiratory rates [41]. Other studies have demonstrated that PGC1α regulates mitochondrial biogenesis in a mouse model of ASD [79] and methylation of the PGC1α promoter region was associated with mitochondrial DNA copy number in children with ASD [80]. Interesting, medication and treatments that activate PGC1α may have some benefit in ASD. Pioglitazone, an activator of PCG1α and mitochondrial biogenesis, attenuated neuroinflammation and oxidative stress in the propionic acid [81] and valproic acid [82] rat model of ASD and cognitive and behavioral impairments in a maternal immune activation [83] and valproic acid [82] rat model of ASD. Pioglitazone has also been shown to improve ASD symptoms is a small open-label case series [84] and in a single-blind placebo controlled prospective cohort [85] and as an add-on to risperidone in a double-blind placebo-controlled trial[86].

Butyrate is an important short-chain fatty acid produced by the enteric microbiome which has been shown to have many positive effects on gut health as well as metabolism [87]. In an LCL model of mitochondrial dysfunction in ASD, butyrate has been shown to protect the LCLs with high respiratory rates (AD-A) from physiological stress and upregulate genes important in mitochondrial dynamics related to repair and morphology (MFN2, DRP1, FIS1, PINK1), resistance to oxidative (UCP2, SOD2, NRF2) and cellular (PCG1α, HIF1α) stress and mitochondrial biogenesis (SIRT3) [42]. In animal models of ASD butyrate normalized behavior and physiological brain abnormalities [88, 89]. Butyrate has been proposed as one of the links between ASD and imbalances in the microbiome [90].

## Limitations

This study is limited by sample size given the invasive nature of obtaining fibroblasts from children. Furthermore, since mitochondrial function is heterogenous across tissues, result may be specific to fibroblast respiration and further studies will be needed to investigate relationships between mitochondrial morphology and mitochondrial respiration in other tissues.

## Conclusions

This study suggests that variations in mitochondrial morphology of fibroblasts from individuals with ASD are associated with mitochondrial enzyme activity. Interestingly, fibroblasts with a relatively uncoupled respiratory chain have a mitochondrial morphology more akin to that of control fibroblasts, whereas those with a more coupled respiratory chain tend to have a more atypical morphology. The cause-effect relationship between respiratory chain status and mitochondrial morphology is not clear. However, given the fact that multiple lines of research suggest that high levels of oxidative stress are associated with ASD [91] and the fact that uncoupling of the mitochondrial respiratory chain can reduce oxidative stress, we speculate that respiratory chain uncoupling is most likely an adaptive mechanism to maintain mitochondrial health. However, although such an adaptive process might help maintaining a healthy mitochondrial pool, increased proton leak across the inner mitochondrial membrane has been associated with reduced synaptic plasticity, potentially resulting in atypical neurodevelopment. The fact that these pathophysiological abnormalities were associated with the severity of ASD symptoms suggest that, at least for a subset of patents, ASD severity may be on a continuum which could be positively modulated by targeting specific subcellular pathways. Therefore, further research is needed to explore the dynamics of this relationship and how it is phenotypically expressed in the characteristics of ASD.

## Supplementary information

Supplementary Table 1
