# Supplementary material for: Mitochondrial morphology is associated with respiratory chain uncoupling in autism spectrum disorder
Source: Transl Psychiatry. 2021 Oct 13;11:527. doi: 10.1038/s41398-021-01647-6 (PMC8514530; doi:10.1038/s41398-021-01647-6)
Supplement: Supplementary file 1 — Supplementary Table 1 [file 41398_2021_1647_MOESM1_ESM.docx]

**Table S1.** Association between Electron Transport Chain Complex activity and Morphological Measurements in Fibroblasts derived from children with Autism Spectrum Disorder. All parameters were significant at p<0.0001

|  | Model 1 | | | Model 2 | |
| --- | --- | --- | --- | --- | --- |
|  | Complex I+III | Complex IV | R-Squared / R | IV minus I/III | R-Squared / R |
| **Cellular Parameters** | | | | | |
| Area | F(1,17.5)=107.30 | F(1,19.2)=142.06 | 33.7% / 0.58 | F(1,7227.4)=892.38 | 33.7% / 0.58 |
| Max Intensity | F(1,16.4)=64.62 | F(1,17.5)=64.45 | 31.1% / 0.56 | F(1,8014.1)=645.44 | 31.2% / 0.56 |
| Perimeter | F(1,17.4)=116.44 | F(1,19.1)=147.57 | 27.8% / 0.53 | F(1,7219.0)=942.90 | 27.8% / 0.53 |
| Compaction | F(1,21.8)=103.92 | F(1,25.8)=103.98 | 12.4% / 0.35 | F(1,4110.5)=393.77 | 12.4% / 0.35 |
| Roundness | F(1,19.0)=100.50 | F(1,21.4)=101.89 | 20.4% / 0.45 | F(1,6151.3)=545.36 | 20.4% / 0.45 |
| **Cluster Parameters** | | | | | |
| Fractal 8 | F(1,19.4)=65.65 | F(1,22.0)=61.82 | 29.6% / 0.54 | F(1,6018.0)=337.47 | 29.6% / 0.54 |
| Fractal 32 | F(1,19.1)=66.05 | F(1,21.4)=65.52 | 32.0% / 0.57 | F(1,6216.6)=369.34 | 32.0% / 0.56 |
| Fractal 64 | F(1,18.5)=69.49 | F(1,20.66)=71.85 | 34.8% / 0.59 | F(1,6606.0)=432.14 | 34.8% / 0.59 |
| Count | F(1,22.0)=63.78 | F(1,25.9)=88.37 | 21.0% / 0.46 | F(1,3989.3)=294.25 | 21.0% / 0.46 |
| Area | F(1,20.3)=30.68 | F(1,23.3)=25.52 | 44.9% / 0.67 | F(1,5240.7)=137.06 | 44.9% / 0.67 |
| Compaction | F(1,27.7)=72.51 | F(1,34.5)=65.95 | 26.5% / 0.51 | F(1,2338.0)=183.54 | 26.5% / 0.51 |
| Elongation | F(1,25.1)=78.28 | F(1,30.6)=82.47 | 32.3% / 0.57 | F(1,3131.8)=252.66 | 32.3% / 0.57 |
| Roundness | F(1,27.1)=42.88 | F(1,33.2)=30.80 | 34.0% / 0.58 | F(1,2879.1)=106.72 | 34.0% / 0.58 |
| Mean Intensity | F(1,231.3)= 11.39 |  | 30.8% / 0.56 | F(1,5914.5)=11.52 | 30.8% / 0.56 |
| Max Intensity | F(1,382.8)=46.89 |  | 30.0% / 0.55 | F(1,6364.0)=50.76 | 30.1% / 0.55 |
| Perimeter | F(1,20.2)=25.41 | F(1,23.1)=19.34 | 45.0% / 0.67 | F(1,5303.4)=109.13 | 45.0% / 0.67 |
| Solidity | F(1,171.0)=11.90 |  | 36.8% / 0.61 | F(1,4002.9)=12.93 | 36.8% / 0.61 |
| **Skeletonized Parameters** | | | | | |
| Skeletal Width | F(1,20.6)=40.85 | F(1,23.6)=51.65 | 45.8% / 0.68 | F(1,4967.3)=222.91 | 45.8% / 0.68 |
| Skeletal Length | F(1,25.1)=68.70 | F(1,30.6)=81.91 | 18.2% / 0.43 | F(1,2774.0)=235.25 | 18.2% / 0.43 |
| Branch Points | F(1,24.4)=51.06 | F(1,29.5)=64.38 | 18.7% / 0.43 | F(1,3046.3)=191.62 | 18.7% / 0.43 |
| End Points | F(1,22.9)=60.33 | F(1,27.2)=81.00 | 20.0% / 0.45 | F(1,3602.3)=256.57 | 20.0% / 0.45 |
| **Isolated Mitochondria** | | | | | |
| Compaction | F(1,27.6)=23.64 | F(1,34.2)=19.05 | 23.4% / 0.48 | F(1,2176.4)=57.43 | 23.5% / 0.48 |
| Elongation | F(1,22.1)=68.70 | F(1,26.0)=71.91 | 37.5% / 0.61 | F(1,4121.7)=274.10 | 37.5% / 0.61 |
| Roundness | F(1,31.1)=37.16 | F(1,39.6)=31.00 | 19.3% / 0.44 | F(1,1383.8)=83.55 | 19.3% / 0.44 |
| Length | F(1,22.9)=65.39 | F(1,27.2)=65.12 | 37.1% / 0.61 | F(1,3724.2)=242.60 | 37.1% / 0.61 |
| **Mitochondrial Location** | | | | | |
| Cell Membrane | F(1,18.2)=29.43 | F(1,20.1)=39.94 | 51.1% / 0.72 | F(1,6784.1)=234.66 | 51.1% / 0.72 |
| Nucleus | F(1,22.2)=100.45 | F(1,14.5)=49.48 | 30.2% / 0.55 | F(1,2922.6)=398.76 | 30.2% / 0.55 |
|  | | | | | |
| Average |  |  |  |  | 30.5% / 0.55 |
